# Effects of Intestinal Microbiota on Pharmacokinetics of Crocin and Crocetin in Male Sprague-Dawley Rats

**DOI:** 10.3390/metabo10110424

**Published:** 2020-10-26

**Authors:** Rajina Shakya, Mahesh R. Nepal, Mi Jeong Kang, Tae Cheon Jeong

**Affiliations:** College of Pharmacy, Yeungnam University, Gyeongsan 38541, Korea; rajina.shakya182@gmail.com (R.S.); maheshnpl10@gmail.com (M.R.N.); mjkang@ynu.ac.kr (M.J.K.)

**Keywords:** intestinal microbiota, metabolism, crocin, crocetin, pharmacokinetics, in vivo

## Abstract

In addition to the hepatic metabolism, the role of intestinal microbiota in drug metabolism has been considered important in the biotransformation of xenobiotics. Crocin and its aglycone, crocetin, isolated from many plants, including the dried stigma of *Crocus sativus* and the fruit of *Gardenia jasminoides*, have been used in treatment of inflammation, cancer, and metabolic disorders. In this study, the effect of intestinal microbiota on the pharmacokinetics of crocin was studied following single oral treatment with 600 mg/kg crocin to male rats pre-treated with a mixture of antibiotics, such as cefadroxil, oxytetracycline, and erythromycin, for three consecutive days. Following crocin treatment, blood, urine, and feces were collected at various time points for evaluating pharmacokinetic characteristics of crocin and crocetin by using LC-MS. Results showed that intestinal absorption of crocin was relatively marginal when compared with that of crocetin, and that crocin metabolism to crocetin by intestinal microbiota would be a critical step for absorption. The present results clearly suggested that the in vivo pharmacological effects of crocin might be considered as the effects by its aglycone, crocetin, mainly, and that the metabolism of glycosidic natural products by intestinal microbiota should be considered to understand their pharmacodynamic actions.

## 1. Introduction

Intestinal microbiota consists of trillions of microorganisms which act as a likelihood endocrine organ for defending against various pathogens [1]. They also play major roles not only in synthesizing vitamins B and K, but also in metabolizing bile acids, sterols, and xenobiotics [2]. Therefore, intestinal microbiota directly and indirectly provides a defense line to host, including the alteration of pharmacological or toxicological effects of numerous drugs and toxicants [3,4]. In fact, various synthetic drugs and natural products containing glycosidic bonds could reportedly be metabolized by a variety of enzymes secreted by intestinal microbiota, but not by the host [5]. Intestinal microbiota contains extensive metabolic capacity, primarily by enzymes for the hydrolysis of glycosidic bonds [6,7]. In addition, enzymes in intestinal contents liberate de-conjugated drug metabolites for the reabsorption of drugs or bioactive metabolites, thereby increasing bioavailability [8]. Despite many studies on microbial importance in xenobiotic biotransformation, the possible role of intestinal microbiota in xenobiotic metabolism has still not been studied extensively.

**Figure 1:**
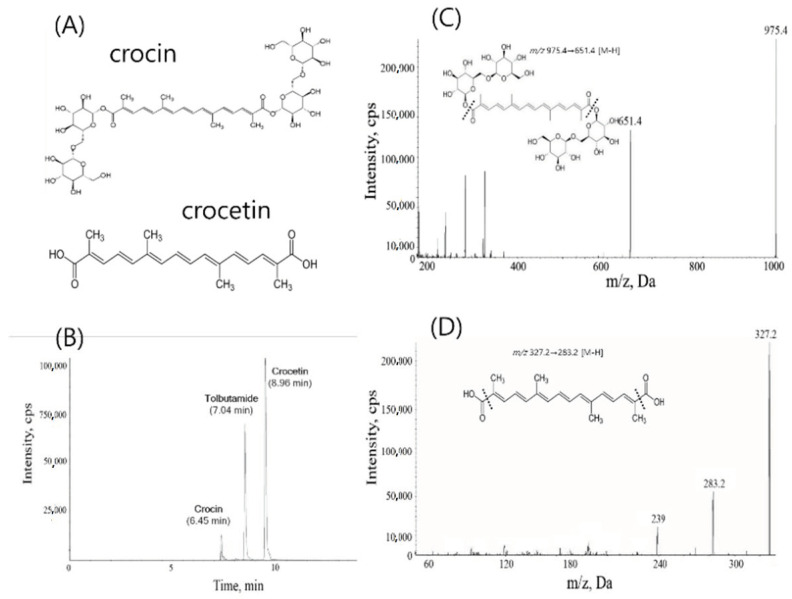
(**A**) Structures of crocin (top) and crocetin (bottom). (**B**) LC/MS chromatograms of crocin, crocetin, and tolbutamide, an internal standard. (**C**) MRM spectrum of crocin. (**D**) MRM spectrum of crocetin.

Crocin, a diester of two disaccharide gentiobiose (Figure 1A), is found in *Crocus sativus* and *Gardenia jasminoides* and appears as deep red crystalline form [9,10]. Crocin possesses various pharmacological properties, such as antioxidant, anti-tumor, memory enhancing, anti-depressant, anti-anxiolytic, and aphrodisiac effects with high efficacy along with no major toxicity [11,12]. Crocetin, the aglycone of crocin containing an apocarotenoid dicarboxylic acid, is found as a brick red crystal, whose structure forms a central core of crocin [13]. Moreover, therapeutic effects of crocetin are reportedly similar to those of crocin [14]. Crocin has been characterized as a free radical scavenger, resulting in its antioxidant activity [15]. Meanwhile, other researchers showed that the oral administration of crocin leads to the formation of crocetin somewhere in gastrointestinal tract [9]. Among the literature, Zhang and colleagues recently found that the metabolic transformation of crocin by intestinal microbiota could protects rats from cerebral ischemia/reperfusion injury [16]. In addition, the authors found that the oral pharmacokinetic profiles of crocetin were similar between normal and pseudo-germ-free animals [16]. These results, along with other literature, suggested a critical role of crocin metabolism by intestinal microbiota in its action [9,16]. Although the possible metabolism of crocin to crocetin by intestinal microbiota was already proposed, a careful study to further characterize whether crocin, crocetin, or both is (are) responsible for the pharmacological action of crocin has not been investigated in vivo, with an emphasis on their pharmacokinetic characteristics in plasma, urine, and feces together. Therefore, the effects of crocin metabolism by intestinal microbiota on pharmacokinetic characteristics of crocin and crocetin were investigated in the present study.

## 2. Results

For the determination of crocin and crocetin, an analytical LC-MS method was established in the present study, as described in the Materials and Methods. With the method, the retention times were observed at 6.45 min for crocin, 7.04 min for tolbutamide (an internal standard), and 8.96 min for crocetin (Figure 1B). The LLOQs of crocin and crocetin were 50 ng/mL and 10 ng/mL, respectively, with the CV less than 15%. In LC/MS, crocin and crocetin were detected in the negative ion mode, and mass transitions used in the analysis were *m*/*z* 975.4 → 651.4 for crocin and *m*/*z* 327.2 → 283.2 for crocetin (Figure 1C,D). The mass transition of tolbutamide was *m*/*z* 269.1 → 169.9 (data not shown). Using this method, in vivo pharmacokinetic studies were performed.

**Figure 2:**
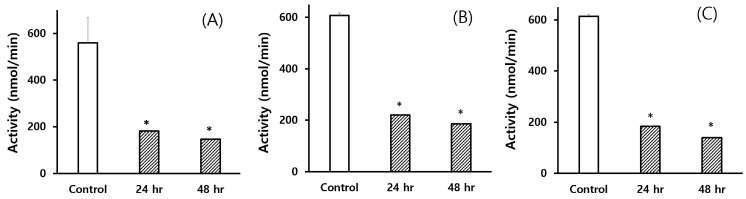
Hydrolytic enzyme activities of the intestinal contents obtained from the rats pre-treated with antibiotics. (**A**) β-Glucosidase, (**B**) β-glucuronidase, and (**C**) sulfatase activities measured by using substrates, p-nitrophenyl-β-d-glucopyranoside, p-nitrophenyl-β-d-glucuronide, and p-nitrophenyl sulfate, respectively. 24 h and 48 h indicate the time of intestinal contents collected after the last treatment with antibiotics. Each bar represents the mean ± S.E. of 3 animals. An asterisk indicates the value significantly different from the corresponding 0-h control at *p* < 0.05 (*).

To ensure that the antibiotic pre-treatment reduced the metabolic capacity of intestinal microbiota, three representative enzyme activities were determined in the intestinal contents prepared from the rats pre-treated with a mixture of three antibiotics. Because most of the enzymes produced by intestinal microbiota are hydrolyzing enzymes, the activities of β-glucosidase, β-glucuronidase, and sulfatase were determined with different substrates in the present study. To determine the appropriate time for crocin administration, the intestinal samples were collected at 24 and 48 h after the last antibiotic treatment (Figure 2A–C). The results clearly showed that the enzyme activities in the intestine could be significantly reduced at both 24 and 48 h after the final administration with antibiotics, suggesting that the present antibiotic pre-treatment model would be appropriate for studying the role of intestinal microbiota in xenobiotic metabolism, and that crocin pharmacokinetics would be investigated at 24 h after the last dose of antibiotics. Based on these results, rats were treated with crocin 1 day after the last dose of antibiotic pre-treatment in the present study.

**Figure 3:**
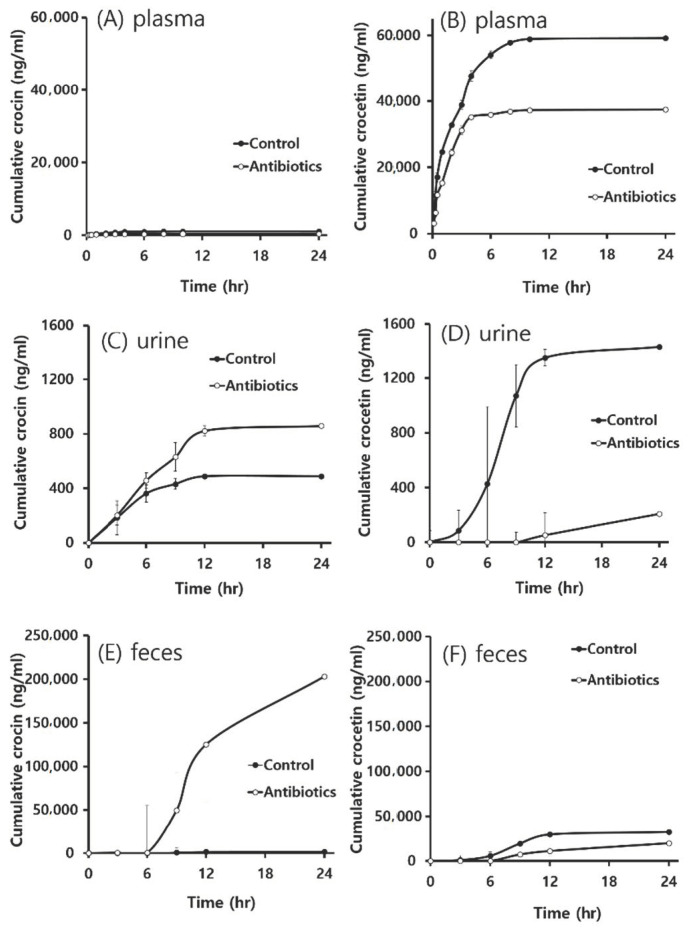
Cumulative concentration versus time profiles of crocin (**A**,**C**,**E**) and crocetin (**B**,**D**,**F**) in plasma (**A**,**B**), urine (**C**,**D**) and feces (**E**,**F**) after single oral administration of rats with 600 mg/kg crocin to control and antibiotic pre-treated rats. Each value represents the mean ± S.E. of 5 animals.

Following the oral treatment with 600 mg/kg crocin to rats pre-treated with a mixture of antibiotics once a day for 3 consecutive days, the concentration of crocin and crocetin produced was determined in blood, urine, and feces at the designated time points to compare the results from control rats (Figure 3). Upon oral administration of rats with crocin, it was negligibly detected in plasma, whereas its aglycone, crocetin, was detected abundantly, indicating that crocin would require intestinal metabolism to crocetin prior to the intestinal absorption (Figure 3A,B). In addition, the cumulative plasma concentration of crocetin in control rats was found to be much higher than that in antibiotic pre-treated rats, suggesting that the suppression of intestinal metabolism of crocin to crocetin by antibiotics could affect the lowered plasma concentration of crocetin (Figure 3A,B). As summarized in Table 1, oral pharmacokinetic parameters in plasma were greatly altered by antibiotic pre-treatment. While the C_max_ of crocetin was marginally decreased in antibiotic pre-treated rats (10,262 ± 2005 ng/L vs. 9732 ± 2371 ng/mL), the area under the curve by time (AUC) of crocetin was decreased to 52% of control in antibiotic pre-treated rats (67,911 ± 7987 ng·h/L vs. 35,104 ± 4144 ng·h/L). In addition, most of the pharmacokinetic parameters studied were significantly changed by antibiotic pre-treatment, suggesting that the microbial metabolism of crocin would be a critical factor to understand pharmacological actions of crocin and crocetin.

The cumulative amounts of crocin and crocetin in urine were determined in the present study (Figure 3C,D). Almost double the amount of crocin was eliminated via urine in antibiotic pre-treated rats than in control rats by 24 h. On the other hand, an approximately 7-fold reduction was seen in the elimination of crocetin in antibiotic pre-treated rats during the same period of time, once again indicating that antibiotic pre-treatment could reduce the formation of crocetin from crocin, and that the excretion of crocetin through urine would be negligible or slow when compared with the amount of crocetin in plasma.

In the present study, crocin and crocetin were also determined in the feces collected for 24 h following the single treatment with crocin to rats pre-treated with antibiotics. As shown in Figure 3E,F, it was found that crocin was present most abundantly in the feces, particularly in antibiotic pre-treated rats, and that the amount of crocetin produced from crocin was higher in control rats than antibiotic pre-treated rats. The results indicated that crocetin production would be affected by intestinal microbiota, and that the feces would be the main elimination route of crocin and crocetin. Since a large amount of crocin was detected in feces in case of antibiotic pre-treated groups, the results suggested that intestinal microbiota might be a crucial factor for crocin metabolism, and that the metabolism of crocin to crocetin would be critically required for absorption.

## 3. Discussion

Like hepatic drug metabolism, intestinal microbiota also plays an important role in xenobiotic metabolism [8]. Particularly, numerous natural products including glycosides can be well hydrolyzed to their aglycones by hydrolytic enzymes only produced by the intestinal microbiota, but not by liver [17]. Therefore, it is of interest to characterize whether the biological effects of those natural products are derived from the hydrolyzed aglycone or from the parent compound, because many times the glycosidic compounds are metabolized to its aglycone form prior to the absorption in the intestine.

To study the possible role of intestinal microbiota in xenobiotic metabolism in vivo, several animal models have been developed. Among them, the simplest model that can be adopted in the laboratory would be the pseudo germ-free model where some antibiotics are orally administered prior to the treatment with the test compound. By treatment of animals with oral antibiotics, the number of intestinal microbiota can be controlled [18]. Thereby, the effects of test compounds in antibiotic-pretreated animals would be compared with the effects in control animals that have normal flora of intestinal microbiota. In the present study, rats were treated with a mixture of three antibiotics for three days to control the numbers of intestinal microbiota without affecting hepatic metabolic capacity as previously published [19]. To confirm the reduction of metabolic capacity of intestinal microbiota by antibiotic pre-treatment, three enzymes produced by the intestinal microbiota, such as β-glucosidase, β-glucuronidase, and sulfatase, were measured, and it was found that the enzyme activities were significantly suppressed by antibiotic pre-treatment. In addition, the antibiotic pre-treatment did not show any toxic signs, including CYP enzymes in liver, suggesting that the animal model we employed for comparing pharmacokinetics of crocin would be appropriate (data not shown).

To further characterize the role of crocin metabolism by intestinal microbiota in its action, the pharmacokinetics of crocin and crocetin following the pretreatment of rats with antibiotics were investigated in plasma, urine, and feces in the present study. Although the dose of crocin was higher than that studied in the original finding, it was not only within the ranges that adopted by other studies but also an intention to determine crocin and crocetin in urine where the amounts were very low [12,16]. When crocin was orally administered to rats pre-treated with antibiotics, the plasma concentration of crocetin, the aglycone form, was significantly less than that in normal rats, suggesting that the intestinal microbiota might play an important role in the metabolism of crocin in intestine. Specifically, the results clearly indicated that large amounts of crocin could be metabolized to crocetin by intestinal microbiota in normal rats than antibiotic-pretreated rats. In addition, less amount of crocetin excretion in urine was observed in the present study, which would be supported in part by a literature that crocetin in plasma might bind to albumin because of the presence of carboxyl group at its both end of hydrocarbon chain [20]. The possible reason(s) for the low excretion of crocetin in urine should be investigated in the near future. Nevertheless, the high concentration of crocetin in plasma indicated that the metabolism of crocin to crocetin would be necessary for the absorption.

Zhang and colleagues observed that crocin was biotransformed to crocetin in large amounts only when crocin was orally administered [21]. The authors also reported that, upon treating the rat with crocin intravenously, no crocetin was observed in plasma, but only crocin was detected, indicating that hepatic metabolism of crocin would be negligible. Recently, Zhang and colleagues also investigated the relationship between intestinal microbiota and crocin by comparing the pharmacokinetics of crocin and crocetin in pseudo-germ free rats, and found that the level of blood crocetin was lowered in the pseudo-germ free group when compared with that of controls, indicating that less amount of crocin was metabolized to crocetin [16]. More importantly, the authors further found that the pharmacokinetic profiles of crocetin orally administered were similar between normal and pseudo-germ-free animals [16]. Similarly, during our study, in both control and antibiotics pre-treated groups with much higher dose of crocin, crocetin was observed in large amounts in plasma in comparision with crocin (Figure 3A,B), suggesting that crocetin might only be produced in intestine by intestinal microbiota prior to the absorption. Of equal importance, in antibiotic-treated rats, the level of crocetin was lower in both urine and feces, indicating that the reduced number of bacteria might retard the metabolism to crocetin (Figure 3C,E). Comparing the amount of crocin in control with that in antibiotic pre-treated rats, crocin was relatively higher in antibiotic pre-treated animals, indicating the lesser metabolism of crocin due to the lower number of microorganisms by antibiotics. In fact, the poor bioavailability of crocin was observed elsewhere [9,16,21].

**Figure 4:**
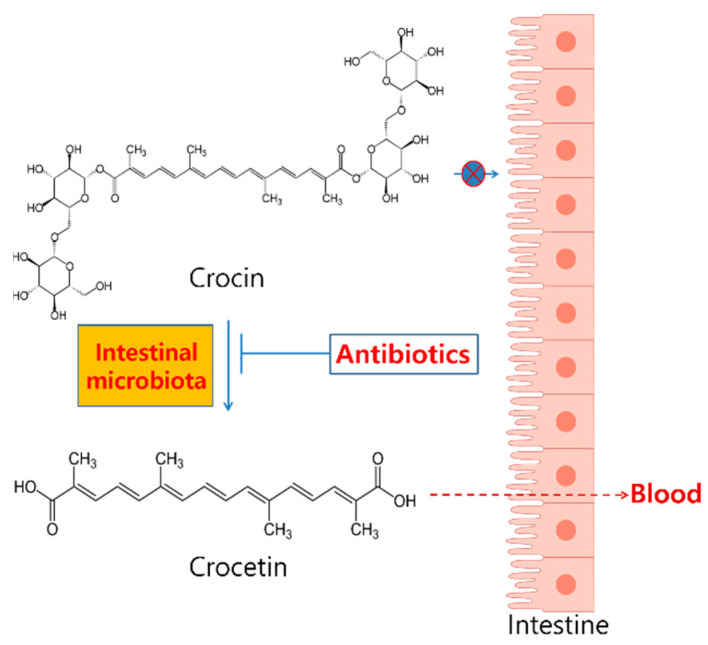
A summary of the role of metabolism of crocin to crocetin by intestinal microbiota.

Although the exact bacterial strains involved in the metabolism of crocin was not demonstrated in the present study, the results clearly indicated that the metabolism of crocin to crocetin by intestinal microbiota might be necessary for the pharmacodynamic actions of crocin (Figure 4). For a better understanding of the role of intestinal microbiota in crocin pharmacokinetics, the identification of specific intestinal microbiota associated with crocin metabolism would be needed in the near future. Taken together, the result from our present study indicated that the in vivo pharmacological actions of orally administered crocin, published elsewhere, would be the activity by crocetin, but not by crocin itself.

## 4. Materials and Methods

### 4.1. Chemicals

Crocin, cefadroxil, oxytetracycline hydrochloride, erythromycin, p-nitrophenyl-β-d-glucuronide, p-nitrophenyl-β-d-glucopyranoside, and p-nitrophenyl sulfate were obtained from Sigma (St. Louis, MO, USA). Crocetin was either prepared from crocin as described below or purchased from ChromaDex (Irvine, CA, USA). Formic acid, acetonitrile, and methanol of HPLC-grades were purchased from J.T. Baker (Central Valley, PA, USA). Potassium monobasic phosphate and potassium dibasic phosphate were purchased from Duksan reagents (Seoul, Korea). All other chemicals were of analytical grades and used as received.

### 4.2. Animals

Seven-week old specific pathogen-free adult male Sprague-Dawley rats weighing 200–300 g were obtained from Samtako Bio Korea (Seoul, Korea). Animals were randomized and housed five per cage. The animal rooms were maintained at: temperature of 22 ± 2 °C, relative humidity of 50 ± 10% with 10–20 air change/h, and light intensity of 150–300 Lux with a 12 h light/dark cycle. Animals used in this study were cared in accordance with the principles outlined in the National Institutes of Health Guide for the Care and Use of Laboratory Animal and were performed with the permission from IACUC at Yeungnam University (approved No. 2014-008).

### 4.3. Animal Treatment

Animals were divided as control vs. antibiotic pre-treated groups. Control groups were orally treated with 10 mL/kg saline, whereas antibiotics pre-treated groups were administered orally with the mixture of cefadroxil (100 mg/kg), erythromycin (300 mg/kg) and oxytetracycline hydrochloride (300 mg/kg) in saline once a day for 3 consecutive days [19,22]. The dosing schedule of antibiotics employed for the present study could effectively control the numbers of intestinal microbiota without showing any possible drug-drug interactions [18,22]. Following the final antibiotic dosing, rats were kept in overnight fast. And then, 600 mg/kg of crocin dissolved in saline was administered orally to both control and antibiotic pre-treated groups. The dose was selected from the literature to clearly characterize the role of crocin metabolism by intestinal microbiota in its pharmacokinetic changes [10]. Blood samples (150 µL) were collected via subclavian vein into heparinized centrifuge tubes at 0, 0.5, 1, 1.5, 2, 3, 4, 6, 8, 10, 12 and 24 h after crocin administration. After centrifugation at 13,250× *g* for 10 min at 4 °C, the plasma was separated and stored at −80 °C until analysis [18]. Urine and feces were also collected at 0, 3, 6, 9, 12, and 24 h after the single crocin administration. Rats were kept in metabolic cages for the collection of urine and feces.

### 4.4. Enzyme Assay

For the study, rats were treated with either saline or antibiotics as mentioned in Section 4.3. The fecal contents, which were extracted from both control and antibiotic pre-treated rats, were mixed with 4 volumes of 0.1 M potassium phosphate buffer, pH 7.4, and were homogenized for 15 s. Fecal suspensions were centrifuged at 500× *g* for 10 min, and supernatants were collected as intestinal contents for the determination of metabolic activities. β-Glucosidase, β-glucuronidase, and sulfatase activities were measured in the present study. For determining enzyme activities, 0.2 mL of intestinal contents was added into the reaction mixture consisting of 0.8 mL of either 1 mM p-nitrophenyl-β-d-glucopyranoside, 1 mM p-nitrophenyl-β-d-glucuronide, or 1 mM p-nitrophenyl sulfate in 0.1 M potassium phosphate buffer, pH 7.4., for preparing the final concentrations of substrates to be 400 μM. The reaction mixture was incubated at 37 °C for 15 min and the reaction was stopped by the addition of 0.5 mL of 0.5 N NaOH. Following the centrifugation at 700× *g* for 10 min, the absorbance of supernatant was measured at 405 nm. A standard calibration curve was prepared with p-nitrophenol [23].

### 4.5. Preparation of Crocetin from Crocin

Crocin was alkaline hydrolyzed to crocetin by 25 mL of 10 N KOH and refluxed at 100–105 °C for 3.5–5 h [24]. After cooling, it was acidified by adding diluted H_2_SO_4_ and the acidification was confirmed using a litmus paper until it turned red. Upon settling of the extract for few h, the sedimentation of crocetin along with a large amount of salt was observed. The upper layer of extract was carefully collected and further processed for benzene extraction. During extraction of crocetin, approximately 20 mL benzene was used each time. Then, benzene extracts were combined and evaporated altogether in a rotary evaporator. The sediment of the extract was then filtered through a filter paper and washed with water. Upon drying, the extract was accumulated in pyridine and vacuum dried conjointly. Subsequently, crocetin was collectively isolated from a preparative TLC method by using hexane-ethyl acetate system with methanol in the ratio of 1:1:0.1 for further purification. The structure of extracted crocetin was confirmed by comparison of its IR and NMR spectral data with those of the commercial crocetin and 93.6% of purity was calculated by an HPLC analysis (data not shown).

### 4.6. Preparation of Samples for Analyses of Crocin and Crocetin in Plasma, Urine and Feces

To quantify crocin and crocetin in plasma, 20 µL of in vivo sample was treated with 100 µL methanol containing 10 ng/mL tolbutamide as an internal standard. For urine, 50 µL sample was treated with 250 µL methanol containing 10 ng/mL tolbutamide. For feces, following a 10-fold dilution with distilled water, 50 µL sample was treated with 250 µL methanol containing 10 ng/mL tolbutamide. Then, the samples were vortexed and centrifuged at 13,250× *g* for 10 min at 4 °C. The resulting supernatant was further passed through a 0.22 µm syringe filter, followed by an injection for the LC/MS analysis. Standard calibration curves were prepared to measure the concentration of either crocin or crocetin against the ratio of the area of either crocin or crocetin to the internal standard.

### 4.7. Analytical Method

For the analysis of crocin and crocetin, an HPLC of Agilent 1260 system with mass spectrometry (API-4000, AB SCIEX) was used. Atlantis dC_18_ column (2.1 × 150 mm, 3 µm, Agilent) was used for separation, and column oven was maintained at 40 °C. The mobile phase was consisted of 0.1% formic acid in water (A) and acetonitrile (B). The stock solutions of crocin and crocetin at 1 mg/mL were prepared in methanol and stored in −80 °C. A linear calibration curve was prepared at a concentration range of 50–10,000 ng/mL for crocin and 10–10,000 ng/mL crocetin for plasma. In case of urine, 0.05–20 μg/mL crocin and crocetin and, in feces, 0.1–50 µg/mL crocin and 0.01–20 µg/mL crocetin were used in LC/MS analyses, respectively. No existence of crocin and crocetin in blank specimen was confirmed at the beginning. Five µL stock solution of either crocin or crocetin were spiked in 45 µL of either plasma, urine, or feces which was extracted in 150 µL methanol containing 10 ng/mL tolbutamide, an internal standard. And then the mixture was further centrifuged and processed for LC/MS analyses in the same condition as described above. The gradient condition for analysis of crocin, crocetin, and tolbutamide was as follows: mobile phase B was started at 10% for 0.5 min, increased to 65% within 1.0 min on hold for 1.0 min, increased to 75% in the next 1.5 min, and increased again to 99% in 0.5 min. The mobile phase B was on hold at 99% for another 3.5 min, returned to 10% in the next 1 min, and was on hold for 6 min to equilibrate the pressure in the column. The flow rate was maintained at 0.25 mL/min during analysis. No endogenous interference was observed in the spiked plasma samples for blank blood samples and data analyses were performed without any corrections, because the matrix effect was negligible (data not shown). Injection volume for analysis was 7.5 µL. Quantitative analysis for crocin and crocetin was performed by multiple reaction monitoring of the precursor ions and the related product ions using the ratio of the area under the peak for each sample.

### 4.8. Pharmacokinetic Parameters

The pharmacokinetic parameters were determined by using the standard non-compartmental method. The pharmacokinetic parameters presented in Table 1 were determined by using WinNonlin (version 1.1, Scientific Consulting).

### 4.9. Statistics

All data were represented as the mean ± standard error (S.E.). Student’s *t*-test was used to compare statistical significance using Sigma Stat 3.5 (Systat Software, San Jose, CA, USA). The significant values at *p* < 0.05 were represented as an asterisk (*).

## Figures and Tables

**Table 1:** Pharmacokinetic parameters of crocin and crocetin following single oral administration to control and antibiotic-treated rats.

Parameters	600 mg/kg Crocin, po			
	Crocin		Crocetin	
	Control	Antibiotic-Treated	Control	Antibiotic-Treated
T_max_ (h)	2.8 ± 1.1	2.5 ± 1.4	3.4 ± 0.9	2.2 ± 0.4
C_max_ (ng/L)	304.4 ± 153.1	74.9 ± 30.1 *	10,262 ± 2005	9732 ± 2371
t_1/2_ (h)	1.8 ± 0.9	15.8 ± 13.7 *	4.2 ± 0.7	3.5 ± 1.9
AUC (ng·h/L)	1251.6 ± 609.1	471.4 ± 448.4 *	67,911 ± 7987	35,104 ± 4144 *
V_d_ (L/kg)	104.8 ± 89.8	0.0 ± 0.0 *	53.6 ± 12.2	84.9 ± 44.0
CL (L/h/kg)	41.4 ± 23.5	0.0 ± 0.0 *	8.7 ± 0.9	17.2 ± 2.3 *

Following the treatment of male SD rats with either saline or antibiotics for 3 consecutive days, rats were treated orally with 600 mg/kg crocin. Each value represents the mean ± S.E. of 5 animals. An asterisk indicates the value significantly different from corresponding controls at *p* < 0.05. T_max_, time of maximum observed plasma concentration; C_max_, maximum plasma concentration; t_1/2,_ terminal half-life; AUC, area in plasma under the curve by time, V_d_, apparent volume of distribution; and CL, apparent total clearance from plasma.
